# Creating a supportive environment for older adults in China ——exploring factors associated with the need for home modifications based on a cross-sectional survey in Central China

**DOI:** 10.1186/s12877-023-04458-0

**Published:** 2023-12-02

**Authors:** Jiajing Li, Bei Wu, Jing Wang

**Affiliations:** 1 School of Architecture and Urban Planning, Beijing University of Civil Engineering and Architecture, Exhibition Hall Road No. 1, Xicheng District, Beijing, China; 2 Rory Meyers College of Nursing, New York University, 433 1st Ave, New York, NY USA; 3 College of Health and Human Services, University of New Hampshire, Hewitt Hall, 4 Library Way, Durham, NH USA

**Keywords:** Home modification, Older adults, Environments, Independence, Need, Cross-sectional survey

## Abstract

**Background:**

A supportive home environment is critical to the safety and quality of life of older adults. Home modification is an effective way to build a supportive home environment for older adults’ aging in place. However, there is a lack of knowledge on older adults’ need for home modifications in China.

**Methods:**

We conducted a cross-sectional survey in three provinces of China (Hubei, Hunan, and Henan) using stratified and cluster sampling methods in 2021. A total of 5485 older adults aged 60 and over were included. The outcome variables were: need for home modifications, level of need, and type of modification needed. Exposure variables included: demographic and socioeconomic characteristics, as well as health conditions. Logistic and Poisson regressions were applied to examine the needs for home modifications and its associated factors.

**Results:**

Nearly 30% of the older adults needed home modifications. The most common choice of home modification was the need for handrails at the bedside, toilet, or threshold (31.64%), and paving un-slip tiles or vinyl flooring (17.45%). Age (IRR = 1.01, *P* < 0.001), education (IRR = 1.11, *P* < 0.01), and level of assistance (IRR = 2.31, *P* < 0.001) were more likely to be positively associated with needs for modification. Participants in the age group of 70 to 79 years, with primary school education, and low-level physically dependent had significantly higher needs for modifications than those of advanced age, lower level of education, or higher level of physically dependent (*p* < 0.01).

**Conclusions:**

The overall need for home modifications in China is low. Home modification programs are needed to tailor individuals’ needs and provide services to those with the most home modification need.

**Supplementary Information:**

The online version contains supplementary material available at 10.1186/s12877-023-04458-0.

## Introduction

Aging in place has been a preferred living arrangement for most older adults worldwide. A rich body of literature shows that home modifications can significantly improve the safety of the home environment and maximize older adults’ level of independence in daily activities [1–3]. In China, 95% of older adults, particularly those with physical limitations, prefer receiving care at home or using day service [4]. However, more than 60% of older adults are living in old houses built more than 20 years ago. In urban area, most of the old house were built in 1970 ~ 90s as welfare house, with relatively low construction standards at that time, the facilities and equipment within them are timeworn and needs modification. While in the rural area, most old houses were self-built with low budget and poor facilities [4]. Unsafe home environments put many Chinese older adults at increased risk of falls and other accidents [5, 6]. Forty million Chinese older adults experience at least one fall per year, and more than half of the accidents happened at home [7]. Therefore, there is an urgent need for China to create a safe and supportive home environment for older adults to better age in place.

In China and other developing countries with rapidly growing aging populations, the importance of home modifications has received increased recognition. Since 2011, the Chinese government launched a series of policies to promote home modifications for older adults [8]. It was also estimated that over 72 cities have launched home modification programs and more than one million families have received home modification services from local government [9]. In the Fourteenth Five-Year Plan of China (2021–2025), providing home modifications for two million families has been listed as a critical target in the national strategy of positively responding to population aging. However, there is a lack of knowledge of older adults’ specific needs regarding home modifications.

Awareness and need for home modification is a critical step towards initiation of home modification [10]. Understanding older adults’ needs and targeting the population with the most urgent need are essential for the government to develop tailored policies with limited public resources. However, there is a paucity of studies on older adults’ need for home modification in China. A few studies found that 40–60% percent of older adults have a need for home modification. However, these studies were conducted using a small sample size, with the targeted study population from big cities [11, 12]. Thus, the current study aims to examine older adults’ needs for home modifications and identify its associated factors using a large-scale survey across urban and rural areas in China.

## Methods

### Sampling and data collection

The current study used a cross-sectional large-scale survey aiming to investigate the wellbeing and daily life of children and older adults in central China. We investigated families with at least one older adult aged 60 years old and over in three provinces (Hubei, Henan, and Hunan). First, we purposely selected 4 rural counties/urban districts representing economic development levels from the three provinces. Second, we randomly selected two rural towns/urban streets from each selected county/district. Third, we selected two rural villages/urban communities from each town/street, which included around 150 households with at least one older adult at the age of 60 years old and over recommended by village/community leaders. Forth, we used a clustered sampling to include all households with at least one older adult at the age of 60 years old and over in the village/community into our survey. A total of 48 urban communities/rural villages including 5494 families with older adults were recruited in the investigation. The survey was conducted from June to October of 2021. The investigators conducted in-person structured interviews with older participants.

### Measures

An interdisciplinary research team developed the questionnaire. The research team involved individuals from diverse fields, including public policy and management, sociology, public health, and law, who worked together to design the questionnaire. It is a systematic process that involves collaboration among researchers from various disciplines, careful consideration of research objectives, construct identification, question generation, pilot testing, and ethical considerations. Questions include older adults’ socio-demographics, family-related information, living arrangement, social security, health conditions, and social support. The questions regarding the living environments and the need for modifications were developed based on a review of literature from PubMed and CNKI databases and policies on home modifications in China [13].

### Outcome variables

Outcome variables were whether there is a need for home modifications, the level of need, and the categories of modification that are needed. Twelve home modification items were included in the survey, which can be divided into five categories, including: (1). Floor modification (paving non-slip tiles or vinyl flooring and floor flattening) (2). Corridor modification (installing indoor threshold ramps, removing threshold, widening doorway) (3). Installing handrails (at the bedside, toilet, and threshold) (4). Appliance modification (power socket and switch modifications, installing automatic sensor nightlight, and changing to lever or sensor faucet) and (5). Bath space modification (removing the bathtub/shower enclosure, installing shower curtain, and expanding the shower space). Participants checking at least one item were identified as having a need for home modification. Participants who chose at least one item in each category were considered as having a need for this modification category. The level of need for home modifications was indicated by the number of modification items that the participants preferred to take.

### Exposure variables

Exposure variables were chosen based on a literature review of studies on the need for home modifications [12–14]. Exposure variables include demographic and socio-economic factors (i.e., age, education level, personal/family annual income), contact with adult children (seldom contact, contact through phone, meeting in face, and other), living arrangement, accessibility of living environment (a proxy of building with an elevator), and health status (self-rated health, disability, and presence of chronic disease). Age was considered as both numerical and categorical variables (60–69, 70–79, 80 and over). Education attainment was classified into three levels (none or less than primary, primary school, and middle school and higher). Related to living arrangements, the question was that “who is living with you in this house/apartment, please list them as my spouse, adult children, or grandchildren”. Participants who listed no one were grouped as those living alone, who listed only spouse/adult children/grandchild were grouped as those living with spouse/adult children/grandchildren, and who listed both adult children and grandchildren were grouped as those living with grandchildren and adult children. Participants who reported good and general were grouped as good health. Barthel Index for Activities of Daily Living (ADL) was used to assess whether the participants were disabled. Items of ADLs, including feeding, bathing, grooming, dressing, toilet use, and mobility on level surfaces, were ranked as independent, needing help, and unable by the participants. We used Barthel Index to calculate the total scores for each participant. We classified participants into four levels of dependence: independent, low-level physically dependent, mid-level physically dependent, and fully dependent.^1^ Participants who reported having at least one type of chronic disease^2^ (14 types of chronic diseases) were grouped as those having chronic diseases.

### Statistical analysis

Multivariated logistic regression was used to explore factors associated with whether older adults need home modifications, as well as factors related to types of need for modification. Poisson regression was applied to identify exposure variables associated with the number of items that needed to be modified. Exposure variables were utilized in part in each analysis. For the existance of a need for home modification,we assumed that it were relatively subjective and can be related to their information accessibility (use of a smartphone), support from family (contact with adult children), and living arrangement. For those who already expressed a need for modification, the level of need and type of need were assumed to be related to their health condition and type of residential building. The return rate was 100% since we conducted a face-to-face investigation to households on the selected lists. Nine questionnaires were excluded because of missing data. All statistical analyses were performed using Stata 14.0 statistical software (StataCorp LLC, College Station, TX, USA).

## Results

### Demographic characteristics

A total of 5485 older adults participated in the survey. As shown in Table 1, the mean age was 71.01(6.29). More than half of the older adults were aged 60–69. Only 12.49% older adults live on their own. The majority of the participants were independent in activities of daily living (ADL) (93.27%). The mean personal annual income was 19156.64 (CNY), ($2962.67) and only 29.54% of the participants identified pension as their main income source.

**Table 1:** Description of characteristics of participants (*N* = 5485)

Variable	Result
**Age in years, mean (SD)**	71.01(6.29)
**Age group, years, n(%)**	
60–69	2,828(51.56%)
70–79	2,042(37.23%)
>80	615(11.21%)
**Personal Annual Income, CNY, mean (SD)**	19156.64(38910.92)
**Main income from pension, N (%)**	
Yes	1620(29.54%)
No	3865(70.46%)
**Education, N (%)**	
None or less than primary	1765(32.18%)
Primary	1646(30.01%)
Middle school and higher	2074(37.81%)
**Contact with adult children, N (%)**	
Seldom contact	65(1.19%)
Contact through phone	3405(62.08%)
Meeting in face	1854(33.80%)
Other	161(2.94%)
**Living arrangement, N (%)**	
Living alone	685(12.49%)
Living with spouse	1737(31.60%)
Living with grandchild(s)	329(6.00%)
Living with adult children	838(15.28%)
Living with grandchild(s) and adult children	1900(34.64%)
**ADL, N (%)**	
Independent	5166(93.27%)
Low-level physically dependent	228(4.16%)
Mid-level physically dependent	92(1.68%)
Fully dependent	49(0.89%)

### Older adults’ need for home modifications

**Fig. 1:**
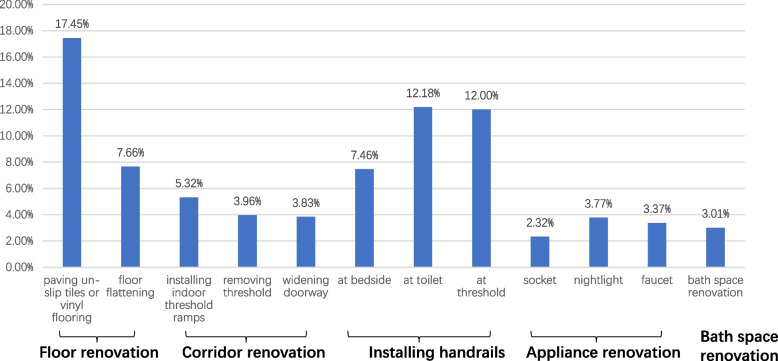
Percentage of need for different types of modification (*N* = 5485)

A total of 29.55% older adults reported a need for home modifications of at least one item on the list. The most frequently identified items included the paving un-slip tiles or vinyl flooring (17.45%), installing handrails at the toilet (12.18%) and installing handrails at the threshold (12%), flattening the floor (7.66%), and installing handrails at the bedside (7.46%). The rest of the items were relatively less considered, ranging from 3.01 ~ 3.96% (See Fig. 1).

### Factors associated with the need for home modifications

We found from the logistic regression model that older adults’ age, personal income, education, living arrangement, and connection with adult children were significantly associated with having a need for home modifications after controlling for covariates such as health-related characteristics (Table 2). The association between age, education, and need for modification was not linear. Participants aged between 70 and 79 had a higher demand for renovating their home environment (*P* < 0.05), while those aged 80 and above did not show a difference in need compared with those aged 60 to 69. Participants with a higher level of personal income expressed less need for modifications (*P* < 0.05). Compared to those with none or less than primary school education, participants with primary school education had 1.24 times of odds of need for modifications (*P* < 0.01), while participants had middle school and higher education showed no significant difference between those who had none or less than primary school education. Contact with adult children was associated with the need for modifications. Those who reached their children by phone (OR = 0.57, *P* < 0.05) or by meeting in person (OR = 0.56, *P* < 0.05) had lower needs than those who seldom contacted their adult children (*P* < 0.05). However, regarding living arrangements, people living with adult children had a higher need for modifications (OR = 1.37, *P* < 0.01) compared with those living alone or with spouses. Also, people living with their grandchild(s) showed less need for modifications (OR = 0.70, *P* < 0.05).

**Table 2:** Logistic regressions on variables associated with the existence of a need for modifications

Exposure variables	Odds ratio(95% CI)
**Age**	
60–69 (ref)	-
70–79	1.16(1.02–1.32)^*^
>80	1.14(0.93–1.40)
**Family Income**	0.99(0.39–0.99)
**Personal Income**	0.99(0.99–0.99) ^*^
**Address consistent with Hukou**:	
No (ref)	-
Yes	1.17(0.95–1.44)
**Education**:	
None or less than primary (ref)	-
Primary	1.24(1.07–1.44) ^**^
Middle school and higher	1.03(0.87–1.21)
**Use of a smart phone**:	
No(ref)	
Yes	0.92(0.81–1.06)
**Contact with adult children**:	
Seldom contact (ref)	-
Contact through phone	0.57(0.34–0.96) ^*^
Meeting in face	0.56(0.34–0.93) ^*^
Other	0.61(0.33–1.11)
**Living arrangement**:	
Living alone (ref)	-
Living with spouse	1.06(0.87–1.30)
Living with grandchild(s)	0.70(0.51–0.96) ^*^
Living with adult children	1.37(1.10–1.72) ^**^
Living with grandchild(s) and adult children	1.16(0.95–1.43)

*CI* Confidence interval

^*^P < 0.05

^**^P < 0.01

The level of need for home modifications is represented by the number of items chosen by participants. In the Poisson regression model, most exposure variables showed a significant relationship with the level of need for modifications (Table 3). Although the IRR was closed to 1, participants who were older (IRR = 1.01, *P* < 0.001) and with lower income (IRR = 0.99, *P* < 0.01) tended to choose more items to be modified. Participants with primary school education had a significantly higher need than those with a lower level of education (IRR = 1.11, *P* < 0.01). Participants with better self-reported health status had a lower level of need for modifications (IRR = 0.78, *P* < 0.001), while those with at least one chronic disease tended to have more needs (IRR = 1.16, *P* < 0.001). Regarding the level of assistance, older adults with physical impairment had 2.31 times more need than the functionally independent ones (*P* < 0.001). However, the level of need did not increase with the level of assistance. Participants with partial dependence and complete dependence showed less need than those who were low-level physically dependent (*P* < 0.001).

**Table 3:** Poisson regressions on variables associated with the number of items that needed to be modified

Exposure variables	IRR (95% CI)
**Age**	1.01(1.00-1.01) ^***^
**Personal Income**	0.99(0.99–0.99) ^**^
**Education**:	
None or less than primary (ref)	-
Primary	1.11(1.09–1.19) ^**^
Middle school and higher	1.03(0.96–1.12)
**Building with an elevator**	
No (ref)	-
Yes	1.14(1.07–1.21) ^***^
**Self-rated health condition**	
Poor (ref)	-
Good	0.78(0.73–0.84) ^***^
**Chronic disease**	
No (ref)	-
Yes	1.16(1.09–1.25) ^***^
**Level of assistance**	
Independent (ref)	-
Low-level physically dependent	2.31(2.09–2.56) ^***^
Mid-level physically dependent	2.20(1.89–2.56) ^***^
Fully dependent	1.99(1.61–2.47) ^***^

*IRR* Incidence rate ratio, *CI* Confidence interval

^**^P < 0.01

^***^P < 0.001

To explore factors that influence types of need for modifications, we conducted logistic regressions for each modification category. Age, education, types of building, self-rated health condition, level of assistance, and mental health treatment showed various associations with different types of modifications (Table 4). Age showed a significant relationship only to the need for installing handrails. The relationship between the level of assistance and the need for modification was complicated. Compared to independent individuals, those with a higher level of assistance generally had higher needs (except for corridor modification). Older adults who were low-level physically dependent had 2.54 times the odds of independent ones of floor modification (*P* < 0.001), and mid-level physically dependent ones had 2.73 times (*P* < 0.001). However, for appliance modification, mid-level physically dependent older adults had 2.63 times the odds of independent ones (*P* < 0.01), while low-level physically dependent ones had 1.66 times (*P* < 0.05). Interestingly, in terms of installing handrails, low-level physically dependent older adults (OR = 3.16, *P* < 0.001) and fully dependent older adults (OR = 3.09, *P* < 0.001) showed higher need than mid-level physically dependent older adults (OR = 2.63, *P* < 0.001). Only low-level physically dependent older adults showed a significantly higher need for bath space modification compared to independent ones (OR = 2.80, *P* < 0.001).

**Table 4:** Logistic regressions on variables associated with the existence of a need for different types of modifications

Type of modifications	Floor modification	Corridor modification	Installing handrails	Appliance modification	Bath space modification
Exposure Variables	OR(95% CI)	OR(95% CI)	OR(95% CI)	OR(95% CI)	OR(95% CI)
**Age**	1.01(0.99–1.02)	1.01(0.99–1.02)	1.02(1.01–1.03) ^***^	1.01(0.99–1.03)	1.00(0.97–1.02)
**Family Income**	1.00(1.00–1.00)	1(1.00–1.00)	1.00(1.00–1.00)	1.00(1.00–1.00)	1.00(1.00–1.00)
**Personal Income**	1.00(1.00–1.00)	1(1.00–1.00)	1.00(1.00–1.00)	1.00(1.00–1.00)	1.00(1.00–1.00)
**Address consistent with Hukou**:					
No(ref)	-	-	-	-	-
Yes	1.14(0.90–1.45)	1.21(0.85–1.73)	1.05(0.83–1.34)	1.14(0.76–1.71)	1.44(0.75–2.78)
**Education**:					
None or less than primary (ref)	-	-	-	-	-
Primary	1.31(1.11–1.55)^**^	0.88(0.70–1.11)	1.25(1.06–1.49) ^**^	1.46(1.10–1.93) ^**^	0.79(0.53–1.16)
Middle school and higher	1.14(0.96–1.36)	0.89(0.70–1.14)	1.07(0.89–1.29)	1.37(1.01–1.86) ^*^	0.87(0.57–1.32)
**Building with an elevator**					
No(ref)	-	-	-	-	-
Yes	1.25(1.09–1.44)^**^	1.54(1.27–1.88) ^***^	1.21(1.04–1.40) ^**^	1.02(0.80–1.29)	1.12(0.80–1.57)
**Self-rated health condition**					
Poor (ref)	-	-	-	-	-
Good	0.79(0.68–0.92)^**^	0.70(0.55–0.88) ^**^	0.80(0.68–0.94) ^**^	0.85(0.65–1.11)	0.67(0.45–1.01)
**Chronic disease**					
No(ref)	-	-	-	-	-
Yes	1.24(1.07–1.45)^**^	1.24(1.00-1.54)	1.32(1.13–1.55) ^**^	1.17(0.90–1.52)	1.36(0.92-2.00)
**Level of assistance**					
Independent (ref)	-	-	-	-	-
Low-level physically dependent	2.54(1.92–3.36)^***^	3.08(2.22–4.27) ^***^	3.16(2.39–4.18) ^***^	1.66(1.05–2.60) ^*^	2.80(1.69–4.64) ^***^
Mid-level physically dependent	2.73(1.78–4.20)^***^	4.07(2.56–6.47) ^***^	2.63(1.71–4.01) ^***^	2.63(1.46–4.75) ^**^	1.19(0.46–3.16)
Fully dependent	1.52(0.82–2.83)	3.43(1.80–6.55) ^***^	3.09(1.73–5.52) ^***^	0.52(0.13–2.25)	0.522(0.07–3.91)

*IRR* Incidence rate ratio, *CI *confidence interval

^*^P < 0.05

^**^P < 0.01

^***^P < 0.001

## Discussion

This study provided empirical evidence on older adults’ need for home modifications in China and related factors. The overall need for modifications was low and the most urgent need was to improve environmental safety (floor modification and installing handrails). Age, education, income, and connection with adult children had a strong association with the need for modification. The level of need for modifications was significantly related to age, education, building type, health condition, and level of assistance. Older adults needing different levels of assistance showed varied needs for different types of modifications.

### Low needs for home modifications

Our study showed that the percentage of older adults with a need for home modifications was 29.55%. This percentage is relatively low compared to other developing or developed countries. For example, in Singapore, a developed country with a similar percentage of the aging population to China, 70% of older adults expressed a need for modification [15]. In the United States, 60.7% needed home modifications [16].

The low need for modification reflected in the study may be due to several reasons. First, more than 90% of the participants were independent in ADL. According to our results, independent participates expressed lower level of need for modification. According to Pynoos [10], older adults balance four factors when making decisions for home modifications: perceived susceptibility, perceived severity, perceived efficacy, and perceived cost. Having a need for home modification means being aware of the inconvenience and incapacitation caused by unsafe environments, which independent older adults may not be aware of. Secondly, since most older adults in the rural area of China are self-employed and do not have pensions, it can be inferred that nearly 70% of participants in our study are from rural areas. As previous study showed that 40–60% percent of older adults in big cities has a need for home modification [11, 12], the awareness and availability of information for age-friendly environment may be lower for older adults in rurual areas, resulting in lower need for home modification. Furthermore, according to the investigators’ field notes, in the interview, many participants expressed concern about the cost of the modifications, like making the look of their home becomes unattractive, especially those who live in a house owned by adult children. As stated in the introdcution, poor environment (e.g., 27.5% having no indoor toilet; 32.7% having not bath space or facility) also makes it difficult to conduct modifications [4].

In Japan, Germany, and Singapore, where government-funded home modification programs are well developed, older adults are required to apply for the service by themselves. Therefore, being able to identify the need for home modification is the first step for older adults to improve their home environments for better aging in place. There are many social organizations in these countries, for example, associations for home modification, charities, non-profit organizations, and gerontological research centers in universities, that are playing a critical role in popularizing and promoting home modification.

In China, government-funded home modification program target extremely poor older adults who do not have any income or supporter (like adult children), which only constitute a small percentage of the aging population in China. Falls and other accidents caused by potential risks in home environments may lead to emergency visits, hospitalization, and even disability or death [17]. The caregiving burden and poverty resulting from an unsafe home environment are severe, especially for families in rural areas of China. Thus, it is critical for the government to tailor the support to families with varying levels of resources. For older adults with financial resources, there is a need to promote awareness and knowledge about the benefits of home modification. For those with limited financial resources, covering the cost of home modification should be a part of publicly funded programs, e.g., a part of health insurance [18].

### Types of need for modifications vary between levels of assistance

Regarding the type of need for modifications, fall prevention is the top concern as paving un-slip tiles or vinyl flooring, and installing handrails at the toilet or threshold are among the most popular items chosen by the participants. This result is consistent with research conducted in big cities of China [11]. Empirical research in developed countries found that even basic modifications with low budgets can significantly reduce falls among older adults [6]. Thus, based on the results of the current study, floor modification and installing handrails should be prioritized when the budget for modifications is limited.

Results of the study also suggested that the needs for different types of modifications varied with the level of assistance in a non-linear pattern. In China, many home modification programs funded by the government were offered as a “package” with a standard budget or unified modification items. However, this form of service usually results in a waste of resources or a mismatch between needs and modifications [9].

In developed countries like Sweden, Denmark, Germany, and Japan, policies, programs, and laws on home modifications are mature based on a detailed investigation of needs, and research on the tools, processes, and outcomes of home modifications [19–21]. The modifications are usually personalized based on the results of a detailed assessment of both home environments and the physical conditions of older adults [21]. The results of this study indicate that modification services for each family should be tailored to the different needs of individuals through careful assessment.

### Non-linear relationship between demographic and socioeconomic characteristics and needs for modification

The association between age, education, level of assistance, and needs for modification was not linear. The need for modifications did not increase directly with age. People aged 70–79 expressed the most need. These results are consistent with previous research [12, 22]. In advanced age, it becomes more difficult for frail older adults to move temporarily during the modification and/or adapt to changes in their living environment. The same pattern lies in the relationship between the level of assistance and the level of need for modifications. Low-level physically dependent older adults tend to choose more items for the modification than mid-level physically dependent and fully dependent individuals. It can be inferred that people who need some level of assistance start to perceive their susceptibility and consider their care arrangement, thus having a higher level of need [10]. Although mid-level physically dependent and fully dependent individuals are supposed to experience more inconvenience from a poor environment, the existence of a caregiver may compensate for the difficulties and thus reduce the need for modifications. Also, for fully dependent older adults who are bedridden, the modification of the environment becomes less useful.

Currently, in China, home modification programs funded by the government are usually oriented to poor older adults of advanced age (often over 80), and those who need a high level of assistance. However, our finding indicates that people aged 70–79, who are low-level physically dependent have a higher need for modifications. They may benefit more and adapt better to the modified environment. Policymakers need to redefine the target population of these programs, to make the modification more person-centered.

### Limitations

This study is a cross-sectional survey, therefore it is difficult to establish cause-and-effect relationships between the need for modification and exposure variables. Moreover, we lacked information on some key variables, such as the current home environment of participants, and the place of residence (rural or urban area). Future studies may address differences in the need for modification and quality of home environments between older adults of rural and urban areas. The observed relationships between these variables should be interpreted with caution due to their correlation. The associations may reflect the interconnected nature of these aspects in respondents’ lives. Furthermore, since our recruitment approach relied on recommendations from village and community leaders, there can be a risk of selection bias, for example, older residents with communication difficulties (speech disorder, dementia, hearing impairment, etc.) may not be selected.

## Conclusion and policy implications

This is the first large-scale study focusing on the need for home modifications in China, especially for older adults who live in counties and villages. The overall need for home modifications is low. The relationship between age, education, the level of assistance, and the levels of need for modifications is non-linear. The needs for different types of home modification items vary between levels of dependence. Our findings suggest the need for the dissemination of knowledge on home safety and modifications in China, especially in rural areas. Policies and programs on home modifications should consider the variability of the aging population, especially for middle-aged older adults who were low-level physically dependent, and who expressed more needs for modification in our investigation. Moreover, home modification projects need to be individualized to better meet the different needs of older adults and make the best advantage of public resources.

### Supplementary Information

**Additional file 1.**

**Additional file 2.**

## Data Availability

The datasets generated and analysed during the current study are not publicly available due to participants’ personal and family privacy, but are available from the corresponding author on reasonable request.

## Footnotes

1 The total point is 50. Older audlts scores 0–20 was considered as fully dependent; 21–30 as mid-level physically dependent, 31 − 29 as low-level physically dependent, 50 as independent.

2 The 14 types of chronic diseases include high blood pressure, arthritis, digestive system diseases, heart disease, dyslipidemia, lung disease, diabetes, cancer, liver disease, kidney disease, stroke, mental problems, memory disorders, and asthma.

## References

[1] Oaks M. The importance of home modification for occupational participation and safety for low-income older adult homeowners. 2017 https://sophia.stkate.edu/cgi/viewcontent.cgi?article=1011&context=ma_osot Accessed 11 Nov 2022.

[2] Gillespie LD, Robertson MC, Gillespie WJ, Sherrington C, Gates S, Clemson LM, et al. Interventions for preventing falls in older people living in the community. Cochrane Database Syst Rev. 2012; 2012(9):CD007146. 10.1002/14651858.CD007146.pub3 PMC8095069 22972103

[3] Petersson I, Lilja M, Hammel J, Kottorp A (2008). Impact of home modification services on ability in everyday life for people ageing with disabilities. J Rehabil Med.

[4] Dang J, Wei Y, Liu N (2018). Survey report on the living conditions of China’s urban and rural older persons (2018).

[5] Pynoos J, Steinman BA, Nguyen AQ (2010). Environmental assessment and modification as fall-prevention strategies for older adults. Clin Geriatr Med.

[6] Feldman F, Chaudhury H (2008). Falls and the physical environment: a review and a new multifactorial falls-risk conceptual framework. Can J Occup Ther.

[7] Lu ZM, Wang Y, Ye PP, Er YL, Duan LL (2021). Analysis on epidemiologic characteristics of fall in old people: results from Chinese National Injury Surveillance System, 2015–2018. Chin J Epidemiol.

[8] Notice of the State Council on Printing. and Distributing the “Twelfth Five-Year Plan” for the Development of China’s Aging Care.gov.cn. https://www.gov.cn/gongbao/content/2011/content_1960671.htm. Published 2011. Accessed 3 Sep 2023.

[9] Qin L. Research on the practice framework and method of home modification for the elderly. https://eproxy.lib.tsinghua.edu.cn/http/7VhcqQl8xt08ScP75QpTBoezk4wK1sO3JFpirM5K00mWAjQOTJ/Thesis/Thesis/ThesisSearch/Search_DataDetails.aspx?dbcode=ETDQH&dbid=7&sysid=269467 2021. Accessed 11 Nov 2022.

[10] Pynoo J, Nishita CM (2003). The cost and financing of home modifications in the United States. J Disabil Policy Stud.

[11] Yu Y, Jia S, Tian F, Yu H. Study on elderly-adaptability level of the built residential areas in Shanghai. City Plann Rev. 2017;40(5):20–26.

[12] Wang Y, Cai Q, Li B (2018). The needs and its influence factors for home modification of the elderly over 70 years old living alone: based on the survey conducted in 5 typical Chinese big cities. New Archit..

[13] Ministry of Civil Affairs of the People’s Republic of China. Guiding Opinions on Accelerating the Implementation of the Home Modification Project for the Elderly. (In Chinese)., 2020. http://xxgk.mca.gov.cn:8011/gdnps/pc/content.jsp?mtype=1&id=14078. Accesed 11 Nov 2022.

[14] Freedman VA, Agree EM, U.S. Department of Health and Human Services. Home modifications: Use, cost, and interactions with functioning among near-elderly and older adults. 2008. https://aspe.hhs.gov/reports/home-modifications-use-cost-interactions-functioning-among-near-elderly-older-adults-1. Accessed: 11 Nov 2022.

[15] Yuen B, Lane AP (2019). Adapting public housing to age in place in Singapore. Urban environments for healthy ageing: A Global Perspective.

[16] Meucci MR, Gozalo P, Dosa D, Allen SM (2016). Variation in the presence of simple home modifications of older americans: findings from the National Health and Aging trends Study. J Am Geriatr Soc.

[17] WHO. Falls. 2021. https://www.who.int/news-room/fact-sheets/detail/falls. Accesed 11 Nov 2022.

[18] Szanton SL, Leff B, Wolff JL, Roberts L, Gitlin LN (2016). Home-based care program reduces disability and promotes aging in place. Health Aff.

[19] Makigami K, Pynoos J (2002). The evolution of home modification programs in Japan. Ageing Int.

[20] Russell RC, Marney W, Ian C, Jeremy P, editors. Adaptations without delay: a guide to planning and delivering home adaptations differently. London: Royal College of Occupational Therapists. 2019. https://www.rcot.co.uk/adaptations-without-delay. Accesed 11 Nov 2022.

[21] Pynoos J, Steinman BA, Do Nguyen AQ, Bressette M. Assessing and adapting the home environment to reduce falls and meet the changing capacity of older adults, Environmental Gerontology. Oxon: Routledge; 2013. p. 161 – 79. 10.1080/02763893.2012.673382 PMC6294465 30555202

[22] Wu X, Liu C, Yu J (2017). A Study on the influencing factors of the willingness of home modification. The World Survey Res.

