# Supplementary material for: Creating a supportive environment for older adults in China ——exploring factors associated with the need for home modifications based on a cross-sectional survey in Central China
Source: BMC Geriatr. 2023 Dec 2;23:795. doi: 10.1186/s12877-023-04458-0 (PMC10693095; doi:10.1186/s12877-023-04458-0)
Supplement: Supplementary file 2 — Additional file 2. [file 12877_2023_4458_MOESM2_ESM.docx]

**Questionnaire guide for the study**

For families with elderly people aged 60 and above

1. Age;
2. Income;
3. Education;
4. Hukou;
5. Contact with adult children;
6. Health and medical conditions;
7. Smartphone usage;
8. Living environment;
9. Nursing care need;
10. Demand for nursing homes
